# Semi-Automatic Knowledge Extraction from COVID-19 Scientific Literature: the COKE Project

**DOI:** 10.1093/eurpub/ckac129.643

**Published:** 2022-10-25

**Authors:** D Golinelli, AG Nuzzolese, F Sanmarchi, L Bulla, M Mongiovì, A Gangemi, P Rucci

**Affiliations:** 1 DIBINEM, Department of Biomedical and Neuromotor Science, Bologna, Italy; 2 Research and Policy Unit, AUSL Romagna, Ravenna, Italy; 3 STLab, (ISTC)-CNR, Rome, Italy; 4 STLab, (ISTC)-CNR, Catania, Italy

## Abstract

**Background:**

The COVID-19 pandemic highlighted the importance of rapidly updating scientific information. However, the guidelines’ drafting process is highly time- and resource-consuming. The COKE Project aims to accelerate and streamline the extraction and synthesis of scientific evidence. To do so, the Project used deep learning to implement a semi-automated system that enhances the systematic literature review processes. We aim to show some preliminary results on the automatic classification of abstract sentences in papers related to COVID-19.

**Methods:**

The tool is based on Natural Language Processing algorithms to detect and classify PICO elements and medical terms and organize abstracts accordingly. We built a BERT + bi-LSTM language model. The tool was trained on a corpus of 24,668 abstracts unrelated to COVID-19. We assessed the tool performance in a specific topic related to COVID-19 that has not been covered during training. To carry out manual validation, we randomly selected 50 abstracts. Abstract sentences were classified by 2 domain experts into 7 types: Aim (A), Participants (P), Intervention (I), Outcome (O), Method (M), Results (R), and Conclusion (C). The performance of the tool was compared with that of the experts in terms of precision, recall, and F1.

**Results:**

The classifier proved to have a 76% overall accuracy. Precision, recall, and F1 were above 75% for all types of sentences except I, M, and P.

**Conclusions:**

The results indicate a promising ability of the semi-automated classifier to predict expert-validated labels on abstracts of different topics. Our proposed tool is expected to significantly reduce the effort for producing medical guidelines and therefore have a strong, positive impact, particularly in emergency scenarios. The COKE Project also represents a call-to-action for similar initiatives, aimed at enhancing the information extraction process in medicine.

**Key messages:**

• A rapidly changing healthcare requires fast decisions supported by scientific evidence. This is not compatible with the human limits in cognitive skills that reduce the ability to extract information.

• The COKE Project aims to speed up the creation of healthcare guidelines, semi-automating parts of the workflow, and supporting the human-performed process of extracting and analyzing contents.

